# Supplementary material for: Comparison of Major Protein-Source Foods and Other Food Groups in Meat-Eaters and Non-Meat-Eaters in the EPIC-Oxford Cohort
Source: Nutrients. 2019 Apr 11;11(4):824. doi: 10.3390/nu11040824 (PMC6521004; doi:10.3390/nu11040824)
Supplement: Supplementary file 1 [file nutrients-11-00824-s001.pdf]

**Supplementary Table 1.** Food items within each major protein-source food groups

| **Food group** | **Food items** |
|---|---|
| Red meat | Beef, pork, lamb |
| Processed meat | Bacon/ ham/salami/luncheon meat, sausage, liver |
| Poultry | Chicken/turkey |
| Oily fish | Oily fish |
| Non-oily fish | Fried battered /crumbed fish, white fish, tuna, shellfish, prawns, |
| Legumes/pulses | Hummus, baked beans, other beans or lentils |
| Vegetarian protein alternatives | Tofu, soya burger or sausages, other veggies burgers, quorn/quorn burger, vegan cheese |
| Nuts | Nuts, peanut or other nut butters |
| Cheese | Cheese, cheese sauce |
| Yogurt | Low fat yogurt, full fat yogurt |
| Dairy milk | Full cream milk, semi-skimmed milk, skimmed milk, goat/sheep milk |
| Plant milk | Soya milk with calcium, soya milk without calcium, |
| Eggs | Whole eggs |

**Supplementary Table 2.** Food items within each of the other major food groups

| **Food group** | **Food items** |
|---|---|
| Fruit | Apples, pears, orange satsumas, grapefruit, bananas, other fresh fruit (e.g. grapes), tinned or stewed fruit, dried fruit (e.g. raisins) |
| Vegetables | Tomatoes (raw or cooked), salad vegetables (e.g. lettuce, cucumber), carrots, peas, green beans, broccoli, spring greens, kale, cabbage, Brussels sprouts, cauliflower, spinach, courgettes, marrow, parsnips, turnips, swedes, onion, leeks, mushrooms, sweetcorn, beetroot, coleslaw, avocado, sweet peppers, other vegetables |
| Potatoes – boiled, mashed or jacket | Potatoes-boiled, mashed or jacket |
| Potatoes – fried, roasted | Potatoes-fried, roasted |
| White pasta/noodles | White pasta |
| Wholemeal pasta | Wholemeal pasta |
| Couscous, bulgur wheat | Couscous, bulgur wheat |
| White rice | White rice |
| Brown rice | Brown rice |
| Pizza | Pizza |
| White bread | White bread, and rolls including baguette, pitta, and naan |
| Brown bread | Brown bread and rolls |
| Wholemeal bread | Wholemeal bread and rolls |
| Other bread | Crumpet, tortilla wraps |
| Porridge | Porridge, ready brek |
| Breakfast cereal | All bran, Weetabix, muesli, other breakfast cereal (e.g. cornflakes) |
| Cereal bars | Cereal bar, biscuits (e.g. digestives) |
| Chocolate | Chocolate or sweets, all types |
| Cake | Cakes, pastries, fruit pie, fruit tart, crumble |
| Ice cream | Ice cream, choc ices |
| Milk desserts | Rice pudding, custard, crème caramel, fruit based smoothie, hot chocolate, milkshake, single/double cream |
| Soya dessert | Soya yogurt |
| Crisps | Crisps |
| Tea | Tea, all types |
| Coffee | Coffee, all types |
| Fruit smoothie | Pure fruit juice or smoothie |
| Fruit squash | Fruit squash or cordial |
| Sugar sweetened beverages | Fizzy soft drink (e.g. coke) |
| Diet drinks | Low calories/diet soft drinks |
| Wine and champagne | Wine, champagne, sherry |
| Beer | Beer, lager, or cider |
| Spirits | e.g. whisky |

Not included – sauces, spreads, soups (except nut butter, hummus, and cheese sauce)

**Supplementary Table 3.** Energy intakes from major protein-source foods by diet group

| Major protein source | Diet Group | | | | | |
|---|---|---|---|---|---|---|
| | **Regular-Meat Eaters** | **Low meat-eaters** | **Poultry-Eaters** | **Fish-Eaters** | **Vegetarians** | **Vegans** |
| **Men** | **n=2,852** | **n=880** | **n=65** | **n=782** | **n=1,516** | **n=269** |
| Red meat | 4.8 | 2.5 | 0.0 | 0.0 | 0.0 | 0.1 |
| Processed meat | 2.0 | 1.1 | 0.0 | 0.0 | 0.0 | 0.0 |
| Poultry | 3.5 | 1.2 | 2.4 | 0.0 | - | - |
| Oily fish | 1.4 | 1.6 | 2.0 | 2.0 | 0.0 | 0.1 |
| Non-oily fish | 2.8 | 2.9 | 2.7 | 3.0 | 0.0 | 0.0 |
| Legumes/pulses | 1.3 | 1.6 | 1.6 | 2.0 | 2.3 | 3.5 |
| Vegetarian protein alternatives | 0.3 | 0.9 | 2.0 | 2.9 | 3.6 | 4.9 |
| Nuts | 2.7 | 3.7 | 6.6 | 4.6 | 5.4 | 9.9 |
| Cheese | 2.8 | 3.2 | 3.0 | 4.1 | 4.6 | - |
| Yogurt | 1.6 | 1.7 | 1.8 | 1.9 | 1.6 | 0.0 |
| Dairy milk | 5.7 | 5.7 | 4.4 | 4.6 | 4.1 | 0.4 |
| Plant milk | 0.1 | 0.3 | 0.6 | 0.6 | 1.0 | 3.9 |
| Eggs | 1.4 | 1.5 | 1.2 | 1.6 | 1.4 | 0.0 |
| **Women** | **n=10,145** | **n=3,770** | **n=526** | **n=3,746** | **n=5,156** | **n=532** |
| Red meat | 5.0 | 2.5 | 0.0 | 0.0 | 0.1 | 0.1 |
| Processed meat | 1.7 | 1.0 | 0.0 | 0.0 | 0.0 | 0.0 |
| Poultry | 4.2 | 1.4 | 3.3 | 0.0 | 0.0 | 0.0 |
| Oily fish | 1.7 | 1.8 | 2.3 | 2.1 | 0.1 | 0.1 |
| Non-oily fish | 3.0 | 2.9 | 2.9 | 3.0 | 0.0 | 0.0 |
| Legumes/pulses | 1.3 | 1.7 | 1.9 | 2.1 | 2.4 | 3.7 |
| Vegetarian protein alternatives | 0.4 | 1.0 | 1.9 | 2.7 | 3.6 | 5.3 |
| Nuts | 3.1 | 4.4 | 5.4 | 5.3 | 5.6 | 9.0 |
| Cheese | 2.9 | 3.1 | 3.2 | 4.0 | 4.5 | 0.0 |
| Yogurt | 2.3 | 2.4 | 2.6 | 2.6 | 2.4 | 0.1 |
| Dairy milk | 5.5 | 5.5 | 4.9 | 4.8 | 4.5 | 0.4 |
| Plant milk | 0.2 | 0.4 | 0.6 | 0.7 | 1.0 | 4.6 |
| Eggs | 1.5 | 1.4 | 1.5 | 1.6 | 1.4 | 0.0 |

Values are in % unless otherwise stated. Regular meat eaters were defined as participants who consumed ≥50 grams of total meat (any) per day and low meat eaters were defined as participants who consumed <50 grams of total meat (any) per day. ANOVA was used to compare the mean differences between the diet groups. The *P*-heterogeneity between diet groups was <0.001 for all variables

**Supplementary Table 4.** Pair-wise comparisons for other foods between low and non-meat diet groups with regular meat-eaters*

| | Men | | | | | Women | | | | |
|---|---|---|---|---|---|---|---|---|---|---|
| **Other food groups** | **low meat vs regular meat** | **poultry vs regular meat** | **fish vs regular meat** | **veg vs regular meat** | **vegans vs regular meat** | **low meat vs regular meat** | **poultry vs regular meat** | **fish vs regular meat** | **veg vs regular meat** | **vegans vs regular meat** |
| Fruit | 0.37 | <0.001 | 0.66 | 0.03 | <0.001 | <0.001 | 0.02 | <0.001 | 0.05 | <0.001 |
| Vegetables | 1.00 | 1.00 | <0.001 | <0.001 | <0.001 | 1.00 | <0.001 | <0.001 | <0.001 | <0.001 |
| Potatoes – boiled, mashed or jacket | <0.001 | 0.61 | <0.001 | <0.001 | 1.00 | <0.001 | <0.001 | <0.001 | <0.001 | 1.00 |
| Potatoes – fried, roasted | <0.001 | 0.04 | <0.001 | <0.001 | 0.71 | <0.001 | <0.001 | <0.001 | <0.001 | <0.001 |
| White pasta/noodles | 0.43 | 1.00 | 1.00 | 1.00 | <0.001 | <0.001 | <0.001 | 1.00 | 1.00 | <0.001 |
| Wholemeal pasta | <0.001 | 0.30 | <0.001 | <0.001 | <0.001 | <0.001 | <0.001 | <0.001 | <0.001 | <0.001 |
| Couscous, bulgur wheat | 0.05 | <0.001 | <0.001 | <0.001 | <0.001 | <0.001 | <0.001 | <0.001 | <0.001 | <0.001 |
| White rice | <0.001 | 1.00 | 0.08 | <0.001 | <0.001 | <0.001 | <0.001 | <0.001 | <0.001 | <0.001 |
| Brown rice | 0.01 | <0.001 | <0.001 | <0.001 | <0.001 | <0.001 | <0.001 | <0.001 | <0.001 | <0.001 |
| Pizza | 1.00 | 1.00 | <0.001 | <0.001 | <0.001 | 1.00 | 0.43 | <0.001 | <0.001 | <0.001 |
| White bread | <0.001 | 0.05 | 0.03 | <0.001 | 1.00 | <0.001 | <0.001 | <0.001 | <0.001 | <0.001 |
| Brown bread | 1.00 | 1.00 | 1.00 | 1.00 | 1.00 | 0.50 | 0.43 | 1.00 | 1.00 | 1.00 |
| Wholemeal bread | 0.04 | 0.19 | <0.001 | <0.001 | <0.001 | <0.001 | 0.05 | <0.001 | <0.001 | <0.001 |
| Other bread | 1.00 | 1.00 | 0.04 | <0.001 | 0.09 | 1.00 | 1.00 | <0.001 | <0.001 | <0.001 |
| Porridge | 0.01 | 1.00 | 0.01 | 1.00 | 0.03 | 1.00 | 1.00 | 0.05 | 0.07 | 1.00 |

| | | | | | | | | | | |
|---|---|---|---|---|---|---|---|---|---|---|
| Breakfast cereal | 0.94 | 1.00 | 1.00 | 1.00 | 1.00 | 1.00 | 1.00 | 1.00 | 1.00 | 0.06 |
| Cereal bars | 1.00 | 1.00 | 1.00 | 1.00 | 1.00 | 0.01 | 0.71 | 0.13 | 1.00 | <0.001 |
| Chocolate | 1.00 | 0.40 | 1.00 | 1.00 | 0.67 | 0.52 | 1.00 | <0.001 | 1.00 | 1.00 |
| Cake | 0.35 | 1.00 | 1.00 | 1.00 | <0.001 | <0.001 | <0.001 | <0.001 | <0.001 | <0.001 |
| Ice cream | <0.001 | 1.00 | <0.001 | 0.02 | <0.001 | <0.001 | 0.06 | <0.001 | 0.01 | <0.001 |
| Milk desserts | 0.02 | 0.37 | 0.16 | 0.10 | 1.00 | 0.01 | 0.17 | 0.00 | 1.00 | 0.02 |
| Soya dessert | 1.00 | 0.12 | <0.001 | <0.001 | <0.001 | 0.02 | <0.001 | <0.001 | <0.001 | <0.001 |
| Crisps | 0.14 | 1.00 | <0.001 | 0.01 | 1.00 | 1.00 | 0.16 | 0.01 | 0.63 | 0.38 |
| Tea | 1.00 | 1.00 | 1.00 | 1.00 | 0.04 | 1.00 | 0.14 | 1.00 | <0.001 | <0.001 |
| Coffee | <0.001 | <0.001 | <0.001 | <0.001 | <0.001 | <0.001 | <0.001 | <0.001 | <0.001 | <0.001 |
| Fruit smoothie | 1.00 | 1.00 | 0.05 | 1.00 | 1.00 | 0.24 | 1.00 | 1.00 | <0.001 | <0.001 |
| Fruit squash | 0.02 | 0.94 | <0.001 | <0.001 | 0.05 | <0.001 | 0.29 | <0.001 | <0.001 | 0.17 |
| Sugar sweetened beverages | 0.15 | 1.00 | <0.001 | <0.001 | 1.00 | <0.001 | 0.02 | <0.001 | <0.001 | 1.00 |
| Diet drinks | 0.05 | 1.00 | 0.02 | <0.001 | 1.00 | <0.001 | <0.001 | <0.001 | <0.001 | <0.001 |
| Wine and champagne | 0.05 | 0.07 | 0.08 | <0.001 | <0.001 | <0.001 | <0.001 | 1.00 | <0.001 | <0.001 |
| Beer | 0.06 | 1.00 | 1.00 | 1.00 | 0.03 | 1.00 | 0.12 | 0.59 | 1.00 | 0.34 |
| Spirits | <0.001 | 1.00 | <0.001 | <0.001 | 0.04 | <0.001 | <0.001 | <0.001 | <0.001 | 1.00 |

*All values represent p values for differences between the diet groups that have been corrected with Bonferroni. Regular meat eaters were defined as participants who consumed ≥50 grams of total meat (any) per day and low meat eaters were defined as participants who consumed <50 grams of total meat (any) per day.
